# Retracted: A Fatal Case of Wernicke's Encephalopathy after Sleeve Gastrectomy for Morbid Obesity

**DOI:** 10.1155/2017/6764073

**Published:** 2017-03-15

**Authors:** Case Reports in Surgery

At the request of the authors, the article titled “A Fatal Case of Wernicke's Encephalopathy after Sleeve Gastrectomy for Morbid Obesity” [1] has been retracted. The coroner reviewed the case and postmortem histopathology and concluded that the typical histopathological lesions of Wernicke's encephalopathy were absent, therefore not confirming the initial diagnosis. Reexamination of the patient's file showed that postoperative vitamin B1 levels were within the institutional reference range; no hyperemesis and no neurological signs were revealed. The article stated that “This report is published with the written consent of the patient's family,” but there was misunderstanding and the family did not give explicit permission for publication.
